# Beyond the leptomeningeal angioma: a comprehensive review of MR imaging features of Sturge-Weber Syndrome, from early vascular responses to tissue necrosis

**DOI:** 10.1007/s00247-025-06402-3

**Published:** 2025-10-25

**Authors:** Carmen R. Cerron-Vela, Amirreza Manteghinejad, Savvas Andronikou

**Affiliations:** 1 Department of Radiology, Children’s Hospital of Philadelphia, 3401 Civic Center Boulevard, Philadelphia, PA 19104 USA; 2 Perelman School of Medicine, University of Pennsylvania, 3400 Civic Center Boulevard, Philadelphia, PA 19104 USA

**Keywords:** Sturge Weber syndrome, Neurocutaneous, Early Diagnosis, Diagnostic imaging, Magnetic Resonance Imaging, Brain

## Abstract

Sturge-Weber syndrome (SWS) is a rare congenital neurocutaneous disorder caused by somatic mutations in the *GNAQ* gene, resulting in capillary-venous malformations involving the brain, skin, and eyes. Neurological involvement arises from impaired cortical venous drainage and progressive venous congestion, which can lead to irreversible brain injury characterized by hypoperfusion, gliosis, atrophy, and calcifications. Seizures commonly develop before the age of 2, underscoring the importance of early recognition. Magnetic resonance imaging (MRI) plays a central role in evaluating cerebral involvement in SWS. Although conventional MRI is widely used to identify late-stage features like pial-arachnoid enhancement and cortical atrophy, it also holds potential for detecting early changes when interpreted within the appropriate clinical context. Advanced MRI techniques, including susceptibility-weighted imaging and arterial spin labeling perfusion, provide enhanced sensitivity for identifying early perfusion disturbances and venous anomalies, offering important insights into the progressive pathophysiology of the disease. This review highlights the complementary roles of conventional and advanced MRI techniques in detecting early and evolving imaging features of SWS, pairing imaging findings with its underlying pathophysiology, to support timely diagnosis.

**Graphical Abstract:**

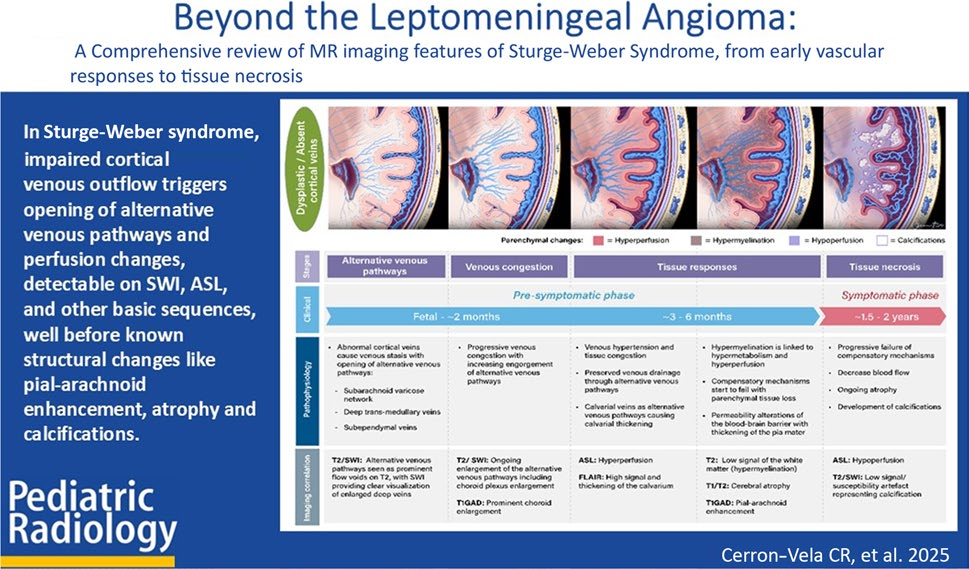

## Introduction

Sturge-Weber syndrome (SWS), also known as encephalotrigeminal angiomatosis [1], is a rare congenital condition marked by cutaneous, neurological, and ocular symptoms, reportedly resulting from capillary-venous malformations affecting the skin, brain, and eyes [2]. It is caused by a somatic mutation (p.Arg183Gln or p.R183Q) in the *GNAQ* gene, which encodes the guanine nucleotide-binding protein G(q) subunit alpha [3]. More recently, *GNA11* variants have been reported to exhibit atypical phenotypes [4].

In the brain, the pathophysiological progression of SWS involves a cascade of neurological changes. Abnormal cortical venous drainage and insufficient deep venous collateral responses lead to cerebral parenchymal venous congestion, triggering a progressive sequence of complications, including hypoperfusion, chronic anoxia, neuronal gliosis, atrophy, and cortical/subcortical calcifications [5, 6].

Most patients with brain involvement in SWS develop seizures by 2 years of age [7, 8], marking a key stage in the condition's progression. The pre-symptomatic phase delimits the opportunity for early treatment or intervention, which could potentially delay or prevent seizures and improve long-term neurological outcomes [8–10].

Neuroimaging is essential for evaluating these patients, with recent advancements such as perfusion MRI using arterial spin labeling (ASL) offering valuable insights into the underlying pathophysiological processes [11, 12]. Additionally, conventional imaging techniques have demonstrated their utility in providing important diagnostic information [13]. For example, Susceptibility Weighted Imaging (SWI) is a 3D, fully velocity-compensated gradient-recalled echo sequence, that exploits deoxyhemoglobin as an intrinsic contrast agent, enabling exquisite non-contrast visualization of small cerebral veins, and longitudinal studies show these medullary veins can enlarge early in SWS [14–16].

Brain involvement in SWS is largely characterized by the formation of what is often referred to as a leptomeningeal or pial "angioma," resulting in chronic brain injury [2]. The term dates to 1935, when angiography showed reduced arterial perfusion and delayed venous drainage that were mistakenly attributed to a leptomeningeal venous mass obstructing venous outflow [5]. Later evidence makes clear this is not a true angioma: as there is no vascular tumor or proliferative growth. Today the term "leptomeningeal angioma" is often used to describe an abnormal vascular plexus on the cerebral surface with corresponding thickening of the leptomeninges, showing post-contrast enhancement on imaging [5, 17]. While it is widely recognized as a key imaging feature for diagnosing brain involvement in SWS, its exact nature was not fully accounted for.

Apart from the so-called “leptomeningeal angioma”, classical imaging descriptions of SWS center around the cerebral atrophy and cortical/sub-cortical calcifications. However, these findings represent late-stage changes in brain tissue that is already irreversibly damaged due to impaired cerebral blood flow. These findings may manifest clinically as seizures, which can further exacerbate the cerebral blood flow disruption [8, 10, 18]. Depending on the extent of brain involvement, stroke and stroke-like episodes can arise [19].

Brain MRI before 2 years of age can detect early physiologic and morphologic changes, which can help with early diagnosis, predict clinical manifestations, and inform management decisions [11, 17, 20–22]. MRI should be focused, in order, on the following: 1) early diagnosis of the primary pathology, which is a lack or paucity of normal cortical veins; 2) detecting the “brain at risk” by demonstrating regions of venous congestion; 3) detecting early tissue responses in the form of engorgement and opening up of alternative venous pathways; 4) detecting intermediate tissue responses such as accelerated myelination; and 5) detecting the tissue sequelae of chronic venous congestion atrophy and calcification.

This review explores the role of conventional and more modern MRI sequences in diagnosing and monitoring pediatric patients with brain-involved SWS, emphasizing early and lesser-known imaging features of the condition identified through these methods and how imaging features reflect the pathophysiology.

## Primary pathology: paucity or lack of cortical veins and opening of alternative venous pathways

Historically, reduced arterial perfusion and prolonged venous drainage times were attributed to the visualized leptomeningeal venous mass, believed to increase resistance to venous outflow [5]. However, abnormal cortical venous development is now considered the central factor in brain involvement in SWS [6]. The absence of adequate superficial venous drainage of the cortex leads to venous stasis and elevated pressure [5]. These changes trigger compensatory enlargement of deep trans medullary venous collaterals and deeper veins, including veins in the choroid plexus, to redirect blood flow toward the deep venous drainage system. These are known as centripetal alternative venous pathways [6, 15, 17, 23]. In this context, the perceived “leptomeningeal angioma” likely represents centrifugally draining enlarged collateral venous channels in the subarachnoid space. These are thought to develop to compensate for an overwhelmed deep venous system as an alternative venous pathway [5, 17, 24].

**Fig. 1:**
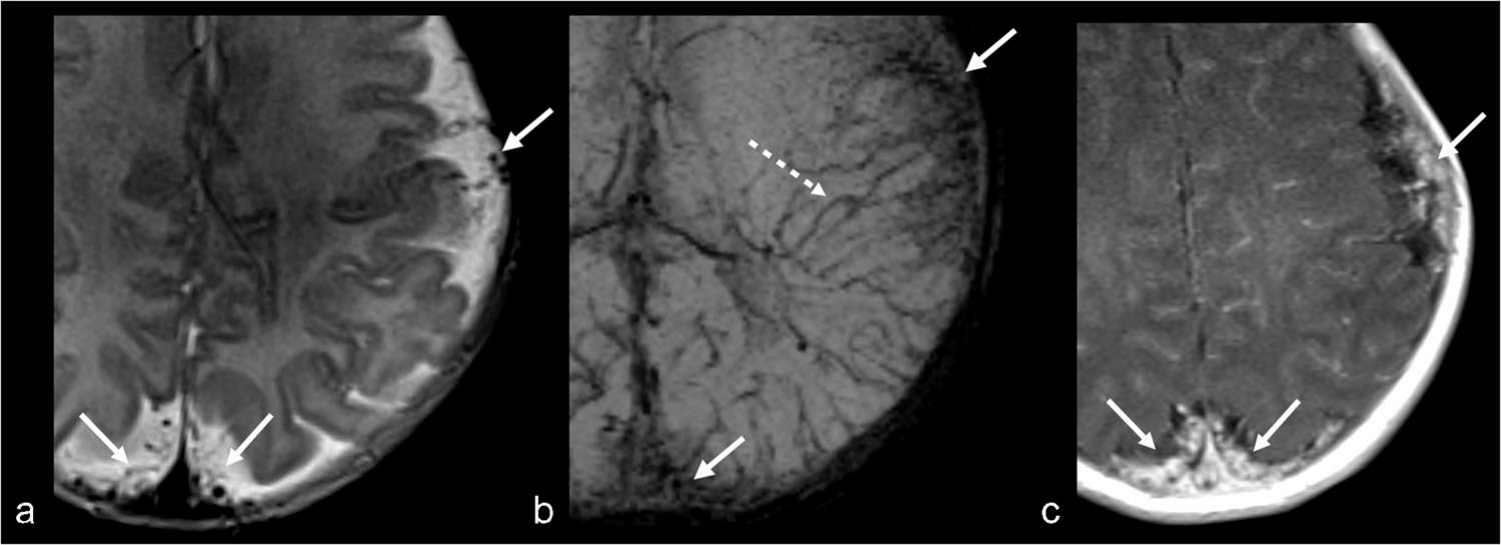
Subarachnoid varicose network without any overt parenchymal changes in an 11-day-old boy with a facial and chest port-wine stain. Axial T2-weighted imaging **(a)** demonstrates the subarachnoid varicose network (*arrows*) as a network of serpiginous flow voids of varying sizes within the subarachnoid space, predominantly on the left, without significant parenchymal abnormality. Axial susceptibility-weighted imaging in minimum intensity projection (SWI MinIP) **(b)**, demonstrates that this is a venous network in the subarachnoid space (*solid arrows*) and is seen in association with prominent trans medullary veins (*dashed arrows*) on the left. Axial T1 turbo spin echo post-contrast image (**c**), shows enhancement of the subarachnoid vascular network (*arrows*) without enhancement of the pial surface of the brain

Venkatakrishna et al. [25] reviewed histopathology specimens from 5 SWS patients who underwent surgery and found no leptomeningeal angiomas, pia mater thickening, or other pia abnormalities. Instead, they identified a vascular network in the subarachnoid space, consisting of venous structures of varying sizes which corresponded to a network of serpiginous flow voids within the cerebrospinal fluid on T2-weighted MRI, that enhanced separately from the pia mater on T1 post-contrast images. These authors introduced a new term to replace “leptomeningeal angioma,” redefining it as a “subarachnoid varicose network.” Others have described these findings as thin-walled small veins and capillaries that penetrate into the depths of the sulci and obliterate the subarachnoid space [26] (Fig. 1). Consistent with this terminology, the entity formerly known as the “leptomeningeal angioma” will hereafter be referred to as *the subarachnoid varicose network*. To emphasize the difference between the subarachnoid varicose network and enhancement of the pia matter, we will use the terminology “pial-arachnoid enhancement” instead of “leptomeningeal enhancement”.

## Early tissue responses: venous congestion and engorged alternative venous drainage

In brain regions affected by SWS, absent or dysplastic cortical veins disrupt the normal drainage of the superficial venous system, resulting in the redirection of venous flow toward the deep venous system, which includes the deep medullary veins. These veins converge near the ventricles and merge with the subependymal veins, forming a characteristic wedge-shaped pattern [15, 27]. The subependymal veins also drain the choroid plexus and deep gray nuclei [28]. This explains why overwhelmed subependymal veins impair the drainage of the choroid plexus, leading to secondary enlargement of the choroid plexus, which can be easily detected on MRI. As described in the previous section, enlargement of deep trans medullary veins and choroid plexus veins constitutes a centripetal alternative venous pathway, whereas the subarachnoid varicose network represents a centrifugal alternative pathway which open as a compensatory mechanism as a compensatory outflow to offload the already congested deep venous system. This is why both enlarged deep venous collaterals and the subarachnoid varicose network can be detected very early in the disease [17].

Some studies have quantified the extension of the enlarged deep medullary veins in SWS and have suggested that the early establishment of effective collateral deep venous flow may reduce the impact of cortical hypoxia, thereby minimizing glutamate-related brain injury and resulting in less severe epilepsy [23].

T2-weighted images not only enable visualization of structural changes like atrophy but can also detect congested cerebral veins, which are clearly observed as prominent curvilinear signal voids [13]. Modern MRI with susceptibility-weighted imaging (SWI) offers superior sensitivity in detecting and evaluating both normal and enlarged deep medullary veins [23, 29]. Recent studies have demonstrated, through SWI data, that small medullary veins can expand during the early stages of SWS [17, 30, 31]. SWI is a 3D, fully velocity-compensated gradient-recalled echo sequence that leverages deoxyhemoglobin as an intrinsic contrast agent, allowing for highly detailed visualization of small cerebral veins without the need for external contrast administration. Although no definitive clinical complications have been linked to contrast administration, the potential risks associated with its use remain uncertain, which makes SWI an attractive imaging technique for demonstrating the venous system [32].

**Fig. 2:**
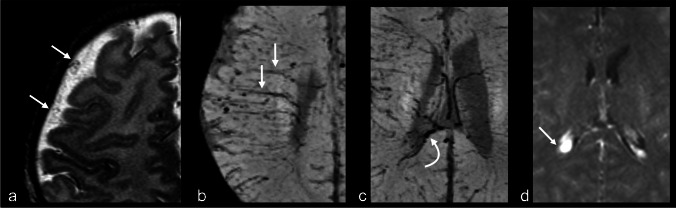
Alternative venous pathways and choroid plexus thickening without any overt parenchymal changes in a 5-week-old girl with a port-wine stain on right side in the V1, V2, and V3 distribution and right eye glaucoma. Axial T2-weighted imaging (**a**) shows the subarachnoid varicose network involving the right frontal lobe (*arrows*). Axial susceptibility-weighted imaging in minimum intensity projection (SWI MinIP) (**b, c**) shows prominence of the trans medullary veins in the right frontal lobes (*straight arrows*) and prominent subependymal veins (*curved arrow*). Axial T1 turbo spin echo post-contrast (**d)** demonstrates right choroid plexus (*arrow*) larger than the left

Other studies have highlighted the importance of identifying dilated alternative venous pathways, particularly the engorgement of the deep venous system, by assessing choroid plexus thickness and its thickness ratio [21, 33, 34]. Catsman-Berrevoets et al. [21] found that measuring choroid plexus thickness on non-contrast MRI is a reliable method for an early diagnosing SWS, with an optimal cut-off value of 5.6 mm achieving 92.9% sensitivity and 100% specificity. This technique was proposed as a safe, non-invasive diagnostic tool for neonates and infants with facial port-wine stains (PWS) and as a complementary approach when contrast MRI is unavailable or does not yet demonstrate pial-arachnoid enhancement. [13] (Fig. 2). Although contrast-enhanced imaging remains a useful tool in the diagnosis of these patients, pial-arachnoid enhancement is not always detectable before 1 year of age and may only become evident thereafter [20, 21, 35]. Recognizing additional key imaging features like assessing for the choroid plexus and the presence of alternative venous pathways, in the appropriate clinical context, support the early diagnosis in these patients.

## Intermediate tissue responses: pial-arachnoid enhancement, accelerated myelination, perfusion changes, and calvarial thickening

### Pial-arachnoid enhancement

Pial-arachnoid enhancement, follows the pial surface of the brain and extends along the sulci and cisterns [36]. The primary mechanism responsible for pial-arachnoid enhancement in general is the disruption of the blood–brain barrier, which is typically linked to infectious meningitis and tumor dissemination, without associated angiogenesis [36–38]. It has been suggested that in patients with SWS, these permeability alterations may result from repetitive and/or prolonged multifocal seizures [39], which are not present in the pre-symptomatic phase and therefore may not present as an early finding. Also, growing evidence suggests that pial-arachnoid enhancement is associated with cortical gray matter tissue loss [40–42]. Similarly, since gray matter tissue loss develops progressively over time in these patients, atrophy and any related pial-arachnoid enhancement may not serve as reliable early diagnostic markers for SWS.

**Fig. 3:**
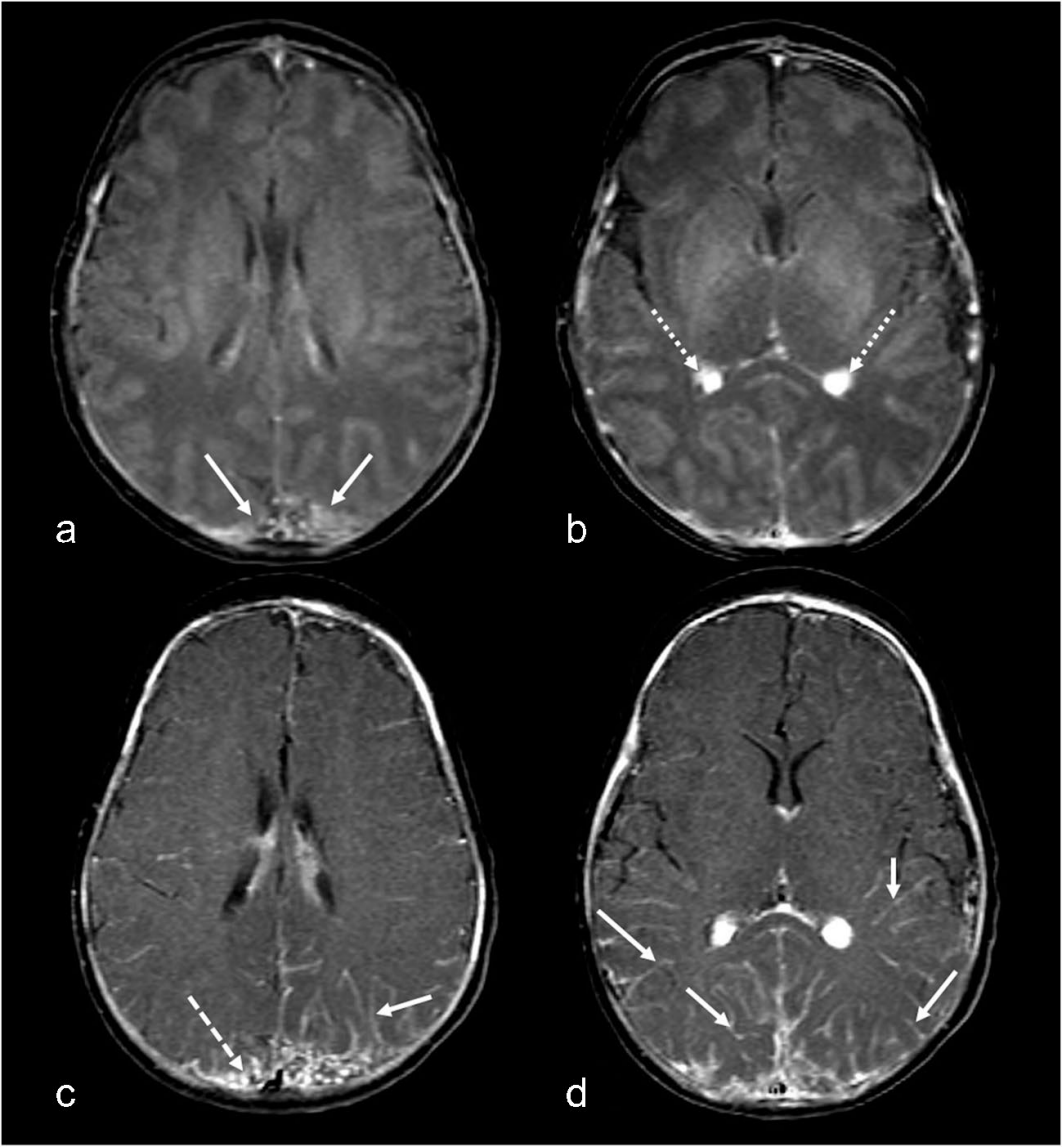
Development of pial-arachnoid enhancement without overt parenchymal changes in a girl with clinical suspicion for Sturge-Weber syndrome. Axial T1 turbo spin echo (TSE) post-contrast at 5 days old (**a, b**) shows enhancement of the subarachnoid varicose network (*solid arrows*), distant from the pial surface of the brain, which is not enhancing. The choroid plexus has a bulky appearance bilaterally (*dashed arrows*). Follow-up axial T1 TSE post-contrast at 3 months old (**c, d**) shows the interval development of bilateral abnormal pial-arachnoid enhancement outlining of the sulci (*solid arrows*) in addition to the distinct and separate enhancement of the subarachnoid varicose network (*dashed arrows*)

In a longitudinal study, Cerron-Vela et al. [17] examined the progression of brain changes in the first 2 years of life in SWS patients. They identified the subarachnoid varicose network on MRI as early as 5 days of age and dilated deep veins at 11 days, while pial-arachnoid enhancement was first noted at 35 days of post-natal life. These findings suggest that pial-arachnoid enhancement is a distinct and more delayed process from the dilation of venous structures within the subarachnoid space which occurs early in the disease. They concluded that alternative venous drainage pathways, particularly the subarachnoid varicose network, frequently precede pial-arachnoid enhancement, highlighting their potential as an earlier imaging marker of brain involvement in SWS. These abnormalities can be identified on MRI sequences that do not require intravenous contrast administration, including T2 and SWI especially using the minimum intensity projection [13] as described previously. (Fig. 3).

Some authors have shown that post-contrast FLAIR imaging suppresses vascular flow, providing a cleaner background for pial arachnoid enhancement. In SWS this can yield a more extensive and definitive depiction of the pial enhancement than post-contrast T1-weighted imaging. [43, 44]. While its role in presymptomatic patients has not yet been evaluated, increased sensitivity of post-contrast FLAIR imaging may offer useful insights in the future.

### Perfusion changes

More modern imaging techniques, such as ASL perfusion MRI, have proven valuable for the early diagnosis of SWS [11, 12, 45]. Venous hypoxia is recognized as the primary insult driving SWS pathophysiology and typically appears as hypoperfusion on imaging. This reduction in cortical blood flow is often associated with a long history of epilepsy and severe brain atrophy [35, 46]. In contrast, hyperperfusion has been identified in younger and asymptomatic patients with SWS that have less brain atrophy [12]. While the exact mechanism driving the increased blood flow remains uncertain, hyperperfusion has been proposed to be associated with impaired blood flow autoregulation during early stages of hypoxic injury [12].

**Fig. 4:**
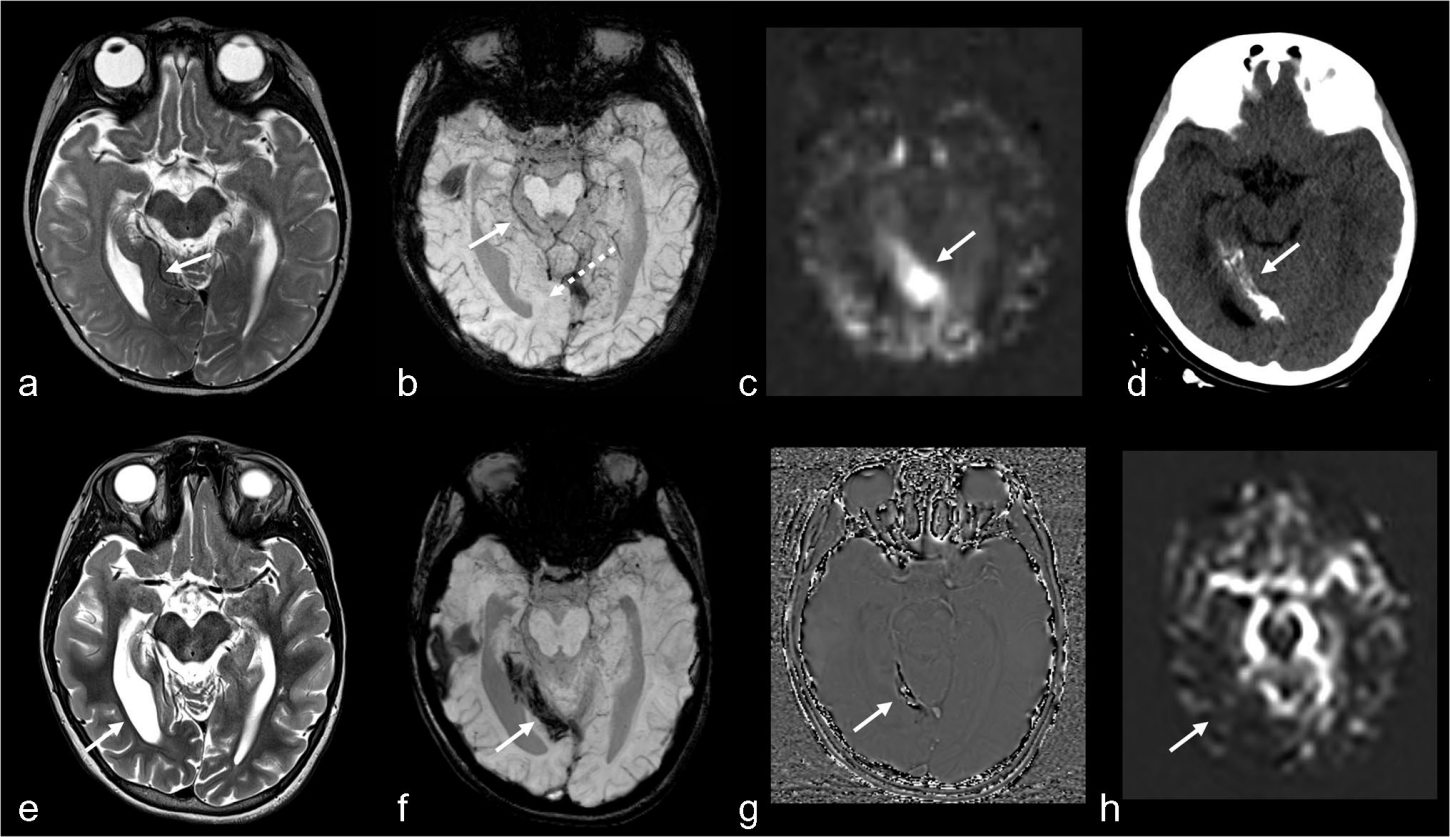
Perfusion changes and evolution to tissue necrosis in a girl presenting with status epilepticus. Magnetic resonance imaging (MRI) at 6 months old **(a-c)**. Axial T2WI (**a)** shows enlarged deep occipital veins tributaries to the internal cerebral vein and vein of Galen. (arrow) without significant parenchymal changes. Axial susceptibility-weighted imaging in minimum intensity projection (SWI MinIP) **(b)** confirms the presence prominent deep veins (solid arrow) and absent cortical veins in the calcarine region (dashed arrow). ASL perfusion **(c)** shows increased perfusion in the medial calcarine aspect of the right occipital lobe (arrow) where there was a lack of cortical veins, indicating venous congestion. Head CT without contrast **(d)** performed at 7 years of age, shows interval development of calcifications, involving in the middle calcarine aspect of the right occipital lobe that show the characteristic tram-track appearance (arrow). Follow-up at MRI at 8-years-of- age **(e–h)** Axial T2 **(e)** shows cortical atrophy with secondary enlargement of the right occipital horn (*arrow*). Axial SWI MinIP **(f)** demonstrates coarse susceptibility artifacts (*arrow*), in keeping with known subcortical calcifications, **(g)** Phase imaging demonstrates signal changes corresponding to calcifications. ASL perfusion **(h)** shows areas of decreased perfusion (arrows), indicating tissue necrosis. Note the hyperperfused (and later hypo perfused areas) matched the location of SWS findings on conventional imaging

Hyperperfusion with normal or near-normal glucose metabolism in the overlying cortex suggests a disease stage where venous drainage is preserved through the deep venous system, compensating for inadequate outflow via the superficial veins [10]. However, as these compensatory mechanisms fail and seizures exert damaging effects, the disease progresses to the classic "burn-out" stage, characterized by hypometabolism, hypoperfusion, and atrophy [11, 18]. This highlights a continuum of metabolic and perfusion changes in SWS, beginning with hyperperfusion due to venous hypertension, followed by hypoperfusion, which is often associated with areas of pial-arachnoid enhancement [45]. These changes occur at varying rates across different brain regions and may not directly correlate with patient age (Fig. 4).

ASL in SWS is timing-sensitive: ictal/early post-ictal hyper perfusion and chronic hypoperfusion can mimic or mask disease. Anesthesia and arterial flow changes which may arise from venous congestion can influence optimal inversion times for ASL [11]. For this reason, we recommend a tailored post label delay (PLD) [11]; and acquire ASL before gadolinium to avoid the “gadolinium effect.”, a phenomenon that occurs when administering a gadolinium-based contrast agent before performing ASL leads to reduced signal intensity, and complete loss of signal from the whole brain [47].

Differential diagnoses for ASL hypoperfusion include vasogenic edema, infection/abscess, and migraine aura. Differential diagnoses for ASL hyperperfusion include "trapped label" (e.g., aneurysm sac), rapid shunting in AVMs, luxury perfusion and migraine during the headache phase [11, 12, 47].

While not a focus of this review, Fluoro-deoxy-D-glucose positron emission tomography (PET) is another valuable tool for assessing the extent of brain involvement in SWS. By measuring cortical glucose metabolism, PET can detect more widespread cortical dysfunction that extends beyond the areas affected by leptomeningeal enhancement [10].

### Hypermyelination

In neonates or young infants without a history of recurrent seizures, hyperperfusion and hypermetabolism are observed in the brain regions underlying the subarachnoid varicose network, which are associated with hypermyelination [48].

Hypermyelination has been linked to transient hyperperfusion and hypermetabolism in the affected region, probably as a stress response to initial hypoxia [48–50]. It has also been documented in association with cerebral venous thrombosis, likely resulting from a state of hyperperfusion caused by venous congestion [49].

**Fig. 5:**
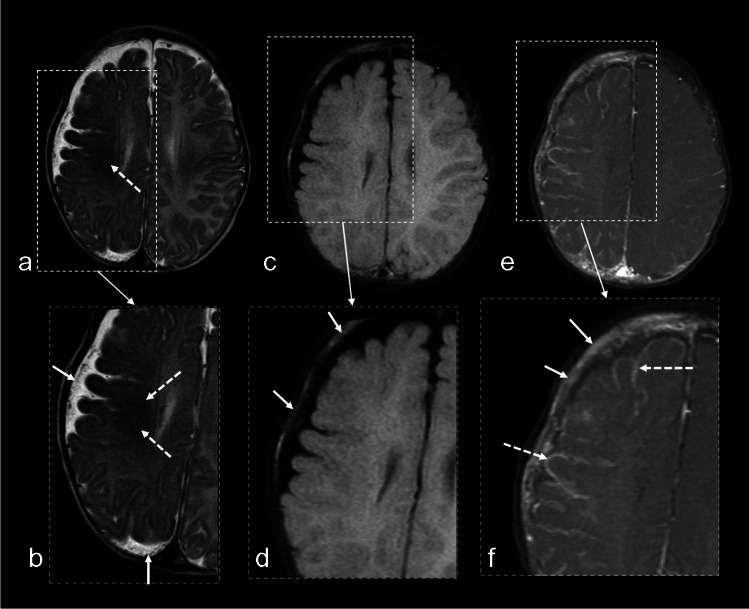
Accelerated myelination and calvarial thickening in a 4-month-old girl with a port-wine stain. Axial T2-weighted imaging **(a)**, with the *rectangle* representing the magnified portion **(b)**, demonstrates decreased T2 signal in the deep white matter of the right cerebral hemisphere (compared to the left and what is expected for age), indicative of accelerated myelination (*dashed arrows*). There is associated atrophy on the ipsilateral side with expansion of the subarachnoid space, accentuating the subarachnoid varicose network (*solid arrows*). Axial fluid-attenuated inversion recovery **(c)**, with the *rectangle* representing the magnified portion **(d),** shows increased signal intensity (*arrows*) and thickening of the calvarium involving the ipsilateral frontal and parietal bones. Axial post-contrast T1 turbon spin echo **(e)**, with the *rectangles* representing the magnified portions **(f)**, demonstrates enhancement of the ipsilateral calvarium (*solid arrows*) as well as extensive pial-arachnoid enhancement on the right side (*dashed arrows*)

Hypermyelination is visualized as (usually asymmetrically) increased T2 hypointensity in the subcortical white matter compared to that expected for age [48]. Accelerated myelination in the involved hemisphere has been recognized as a possible early diagnostic marker, typically identifiable before 6 months of age [49] (Fig. 5).

### Calvarial thickening

The valveless nature of calvarial veins permits shifts in blood flow direction, allowing blood to be shunted into the bone marrow from either intracranial or extracranial compartments. This process can lead to engorgement of marrow vessels in SWS, which act as collateral venous pathways [5, 51]. Consequently, calvarial bone marrow thickening and signal changes have been observed as associated findings in the early stages of SWS and are detectable using basic fluid-attenuated inversion recovery sequences, among others (Fig. 5).

Asymmetric calvarial thickening also occurs in other causes of cerebral hemiatrophy. For instance, in Dyke–Davidoff–Masson syndrome (DDMS) and other perinatal injuries, impaired early brain growth leads to ipsilateral calvarial hypertrophy with paranasal sinus hyperpneumatization [52, 53]. Unlike SWS, these osseous changes are delayed compensation for a remote early insult (absent brain growth fails to counter inward calvarial expansion), and they emerge only when injury occurs before age 3 [53, 54], rather than the early compensatory process characteristic of SWS.

## Late tissue responses: hypoperfusion, atrophy and calcification

The changes from hyperperfusion to hypoperfusion in SWS occur in a continuum representing progression of the disease, culminating in cerebral destruction with calcification and atrophy, when compensatory mechanisms progressively fail [11]. Extensive calcification in the affected hemisphere is linked to significantly reduced perfusion in the underlying white matter and is associated with more severe epilepsy [55]. Cortical or subcortical calcifications (seen best with SWI or computed tomography) and brain atrophy (appreciated best on T2-weighted imaging) are therefore typically absent from MRI scans of neonates or young infants with no history of prior seizures and are not useful imaging features for early diagnosis in SWS [48].

In summary, the above progression is driven by underdeveloped cortical venous outflow, which elevates venous pressure and impairs arterial blood supply to brain tissue. As compensatory mechanisms (i.e., opening up of alternative venous pathways) fail, this ultimately results in hypoperfusion, chronic anoxia, neuronal gliosis, atrophy, and cortical calcification [12, 49, 56].

**Fig. 6:**
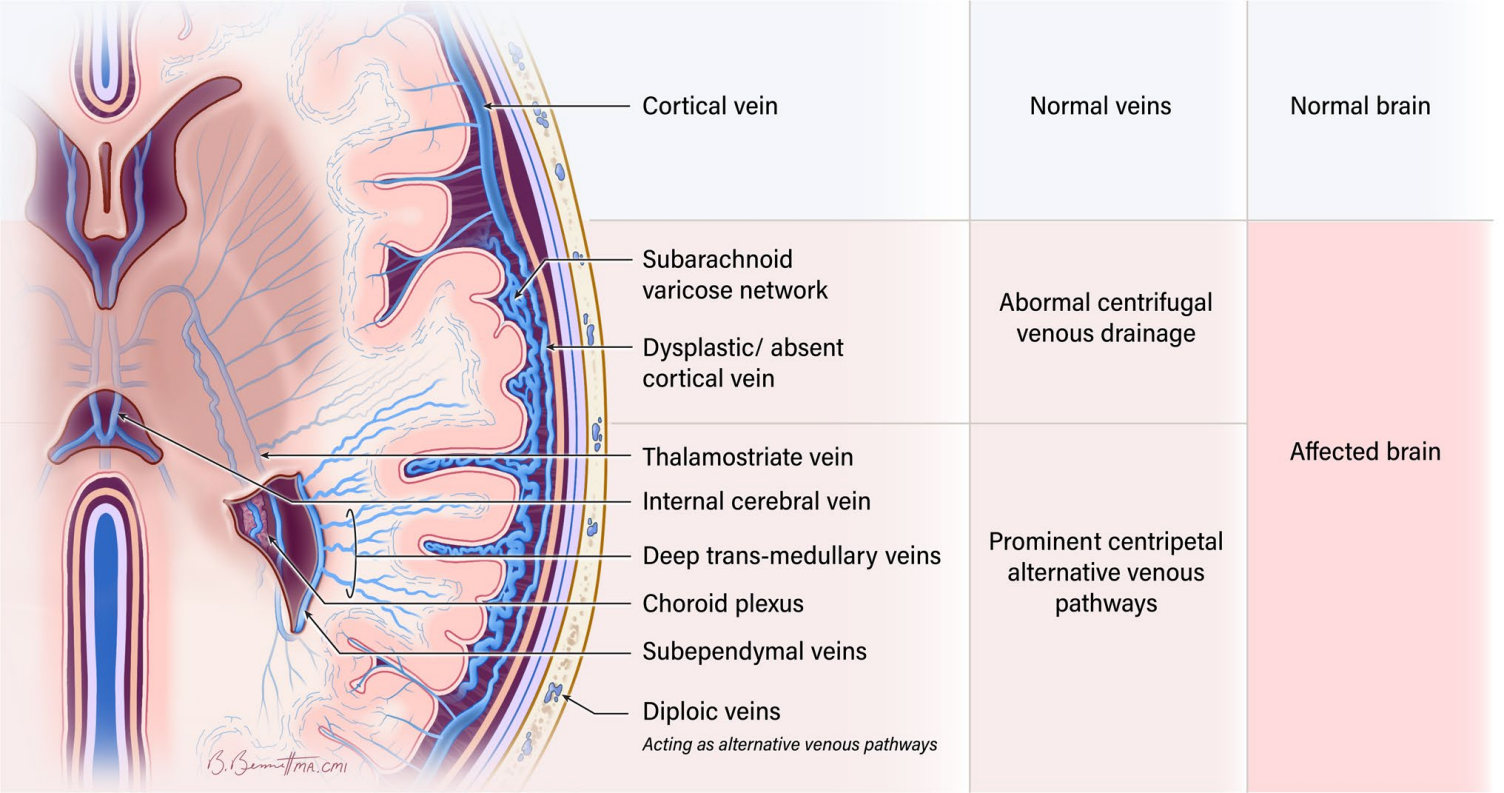
Schematic representation of baseline cerebral venous anatomy and SWS related venous alterations in an asymptomatic patient with intracranial involvement

**Fig. 7:**
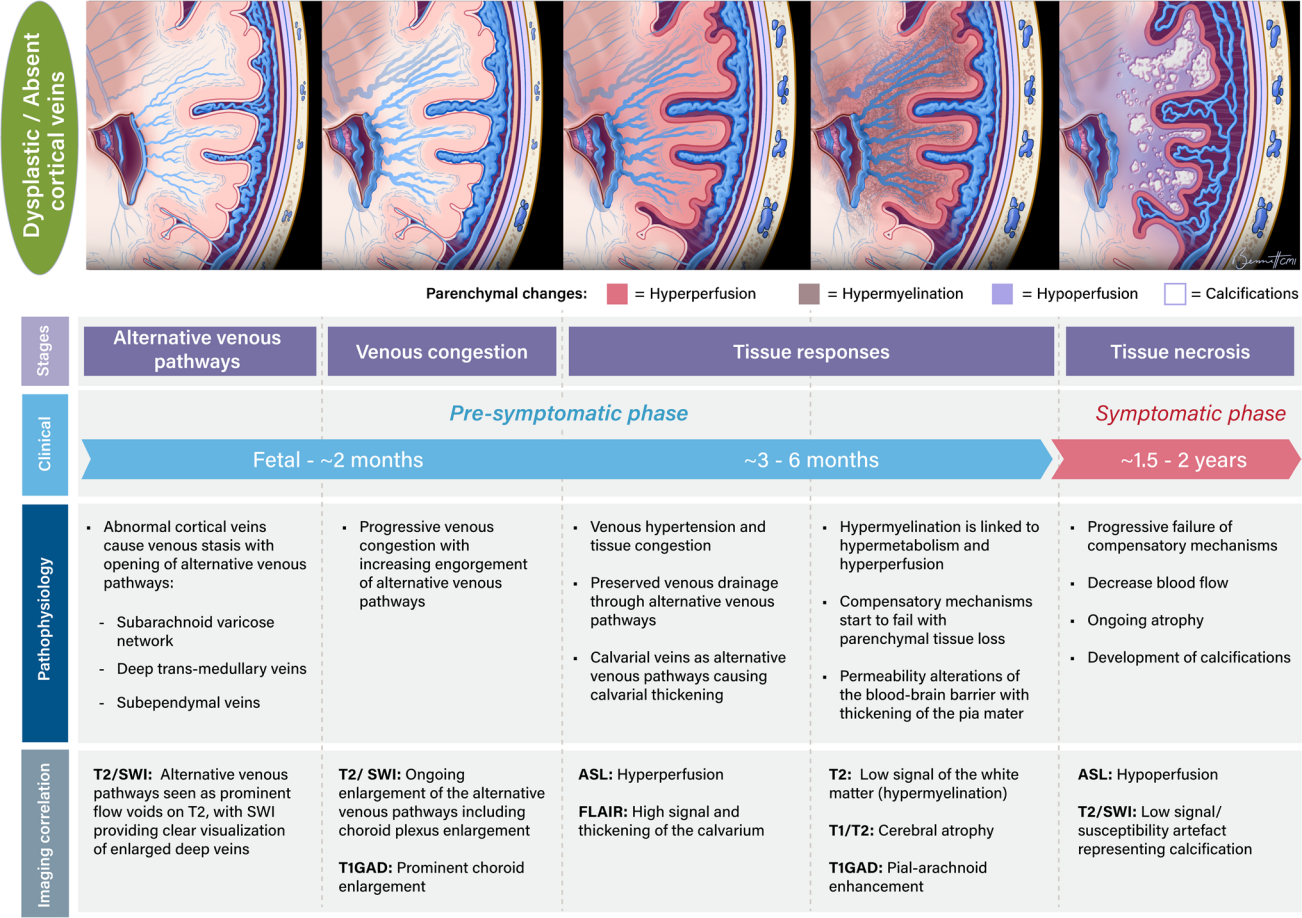
Timeline depicting the evolution of pathophysiological changes, associated imaging findings, and disease stages in SWS with intracranial involvement. Note: post-contrast FLAIR imaging may also be considered for future protocols

In this context, we propose a timeline of imaging findings, grounded in the underlying pathophysiology (Fig. 6 and Fig. 7). This timeline (Fig. 7) is an estimate based on a literature review [8, 10, 11, 17, 45, 57]. Since SWS is a congenital condition, tissue responses may have been present during the fetal and neonatal periods, particularly in severe cases. Milder forms of SWS may follow a different pattern. Some tissue responses also overlap with signs of tissue necrosis. Precisely determining the timing of lesion emergence would require repeated MRIs, which is not feasible.

## Clinical relevance

Early detection of brain abnormalities is particularly important during the pre-symptomatic phase, a critical window for intervention that may delay or even prevent the onset of seizures, ultimately improving neurological outcomes [9]. This phase provides a unique opportunity for pre-symptomatic treatment, which has shown promise in children diagnosed with SWS prior to seizure onset [8]. Presymptomatic treatment for infants with SWS brain involvement was first proposed in 2002 [8, 58]. Since then, small case series using low dose aspirin and antiepileptic drugs suggest delayed seizure onset, but no prospective trials had been completed. [59]. Current consensus favors individualized, expert-centered decisions rather than universal presymptomatic therapy, while stressing the lack of randomized trials [60, 61].

Capillary malformations, such as PWS, and their association with the subarachnoid varicose network (previously called “leptomeningeal angioma”) in SWS highlight a shared developmental mechanism rooted in the primitive venous system's embryological regression failure [62, 63].

The traditional Roach Scale classification divides SWS into three types, using the old terminology of leptomeningeal angiomatosis: Type I (classic Sturge-Weber syndrome), involves both facial and leptomeningeal malformations, often with glaucoma; Type II, characterized by facial capillary malformation alone, which may also present with glaucoma; and Type III, defined by isolated leptomeningeal angiomatosis, usually without glaucoma. [1, 4]. This classification, is now understood to be overly simplistic, particularly regarding type II SWS, as it fails to account for the stratified risk of brain and eye involvement based on the extent and location of facial PWS [4].

The recognition that the risk of brain involvement correlates with the size and location of PWS underscores the importance of identifying high-risk phenotypes [64, 65]. These phenotypes serve as critical markers for early imaging and timely intervention, offering the potential to mitigate complications such as seizures and developmental delay. By prioritizing high-risk cases for early monitoring and treatment, clinicians can adopt a proactive approach that may improve outcomes and reduce the burden of advanced disease [20, 22]. In this context, the early MRI diagnostic findings of SWS such as the paucity of cortical veins, subarachnoid vascular network, and engorged deep medullary veins, all detectable on T2 and SWI and seen well before the development of pial-arachnoid enhancement, are key. Several diagnostic and early intervention algorithms have been proposed to target patients with high-risk PWS, aiming to detect early signs of brain involvement in SWS in pre-symptomatic patients [9, 20, 22], but have largely overlooked alternative venous pathways as the earliest signs of SWS, focusing instead on the post contrast imaging findings. Current evidence indicates two distinct contributors to enhancement in SWS: the subarachnoid varicose venous network, representing a centrifugal collateral venous pathway, and the pial–arachnoid enhancement likely arising from BBB disruption and related mechanisms detailed previously. Accordingly, the term “leptomeningeal angioma” is outdated and should be avoided.

Looking ahead, the recently described early MRI features of SWS would allow and guide novel interventions to restore or support venous drainage to the affected parts of the brain, thereby avoiding or limiting the venous hypertension that currently almost inevitably leads to tissue necrosis.

## Conclusion

Advanced MRI sequences, such as SWI and ASL, enable the detection of early brain changes in SWS, which precede the traditionally described structural changes of pial-arachnoid enhancement, atrophy, and calcification. SWI not only better demonstrates calcification than prior MR sequences but also provides critical detail of alternative compensatory venous pathways in SWS through the detection of deoxygenated hemoglobin. ASL perfusion MRI is useful for demonstrating early brain congestion due to venous stasis, well before it is able to demonstrate hypoperfused brain which may no longer be salvageable. The ability to detect alternative venous pathways and brain congestion at an early stage creates opportunities for intervention before the progression to brain necrosis and calcification, previously considered hallmark diagnostic features of the disease. These modern MRI techniques not only deepen our understanding of SWS pathology and progression but also aid in distinguishing salvageable brain tissue at risk of necrosis, paving the way for more targeted therapeutic approaches.

## Data Availability

No datasets were generated or analysed during the current study.
